# Integrating SOMs and a Bayesian Classifier for Segmenting Diseased Plants in Uncontrolled Environments

**DOI:** 10.1155/2014/214674

**Published:** 2014-11-04

**Authors:** Deny Lizbeth Hernández-Rabadán, Fernando Ramos-Quintana, Julian Guerrero Juk

**Affiliations:** ^1^ITESM, Autopista del Sol, 62790 Xochitepec, MOR, Mexico; ^2^Centro de Investigación en Biotecnología/Laboratorio de Investigaciones Ambientales, Universidad Autónoma del Estado de Morelos, Avenida Universidad 1001, 62209 Cuernavaca, MOR, Mexico

## Abstract

This work presents a methodology that integrates a nonsupervised learning approach (self-organizing map (SOM)) and a supervised one (a Bayesian classifier) for segmenting diseased plants that grow in uncontrolled environments such as greenhouses, wherein the lack of control of illumination and presence of background bring about serious drawbacks. During the training phase two SOMs are used: one that creates color groups of images, which are classified into two groups using *K*-means and labeled as vegetation and nonvegetation by using rules, and a second SOM that corrects classification errors made by the first SOM. Two color histograms are generated from the two color classes and used to estimate the conditional probabilities of the Bayesian classifier. During the testing phase an input image is segmented by the Bayesian classifier and then it is converted into a binary image, wherein contours are extracted and analyzed to recover diseased areas that were incorrectly classified as nonvegetation. The experimental results using the proposed methodology showed better performance than two of the most used color index methods.

## 1. Introduction

Modern agriculture is applying new scientific and technological processes to automate and provide more accurate and appropriate solutions to real problems in agricultural systems [1]. Machine vision represents one of these technological achievements, which is being integrated into the agricultural field because it is considered as accurate and nondestructive and yields consistent results [2]. Machine vision is a feasible sensing technique for plant specific direct applications (PSDA) not only due to its superior spatial resolutions [3], but also for providing numerical attributes of the objects or the scene being imaged [2]. This technology has been developed for many agricultural applications, such as detection of diseases and pests in plants [4–10], weed detection [3, 11–13], and plant species identification [14–16], among others. In particular, the development of vegetation segmentation algorithms from images is a fundamental and complex process in agricultural applications, because it is highly dependent on environmental conditions, which can be controlled or uncontrolled.

Previous studies under controlled environments have proposed a variety of approaches for segmenting diseased areas of leaves before their recognition. Most of them consider a uniform color background and controlled illumination, and, in addition, a single leaf of the plant is commonly analyzed, without considering the overlapping of leaves and the effects caused by the illumination such as shadows, brightness, and highlights, among others [14, 17–19]. The segmentation process of these captured images under such conditions represents nonrelevant complexity and yields efficient results. Many algorithms have been developed for segmenting vegetation, without considering areas of diseased vegetation, from images under uncontrolled environments such as agricultural fields or greenhouses. Such algorithms use commonly color vegetation indices to segment a plant from the background of field images [20], such as the color index of vegetation extraction (CIVE) [21] and the excess green minus excess red (ExG-ExR) [22]; besides color indices, thresholds techniques have also been employed applying, for example, a fixed threshold [23] or the Otsu method [24]; in [25] a more sophisticated algorithm using a mean-shift procedure and a back-propagation neural network (BPNN) was presented and yielded better results than the two methods based on color indices known as CIVE and ExG; Jeon et al. [3] presented an algorithm for segmenting vegetation from a normalized excessive-green image using an adaptive threshold which reported efficient results even under different illuminations.

Owing to the problems mentioned above, not much research has been done on segmenting diseased vegetation in uncontrolled environments. In order to cope with the complexities of the environment and improve vegetation segmentation rates, some methodologies have been proposed. One of them is a methodology combining an unsupervised and a supervised learning method that was proposed by Meunkaewjinda et al. [4] for disease detection in grape leaves, where the grape leaf color was extracted from the background by applying a self-organizing feature map (SOFM) and a back-propagation neural network (BPNN). Even though this work yielded very promising performance, some ambiguous color pixels from the background or the vegetation of the image were incorrectly classified. This problem was due to the fact that the developed algorithms only used the color as input feature, thus reducing the effectiveness of the segmentation algorithms for images captured in natural scenes where objects and the background often exhibit common intensities.

Our research has been developed for tomato greenhouse environments whose illumination and background parameters are considered to be under uncontrolled conditions. Therefore, the task of segmenting vegetation, plants with diseased leaves in our case, is a serious complex task to be carried out.

In this work, the images of tomato plants were captured by a low cost digital camera which produced poor quality images. In the methodology proposed in this paper (hereafter called SEVUE, which stands for segmenting vegetation in uncontrolled environments) the following main processes were developed: an image enhancement process to improve both the quality of the images and the highlight features of interest; a color clustering process using two SOMs, wherein the second SOM refines the results of the first SOM; a classification process using a Bayesian classifier; and the extraction and analysis of contours from a binary image to recover areas that were incorrectly classified as nonvegetation and eliminate noise.

The SEVUE methodology was implemented and compared to the color index methods more commonly used for segmenting vegetation under uncontrolled environments (CIVE and ExG-ExR) using the Wilcoxon signed rank sum test to assess the algorithm performance. The remainder of this paper is organized as follows. In Section 2 the SEVUE methodology is described. The procedure used to assess the algorithm performance is presented in Section 3. A discussion of the results is made in Section 4, and finally the extracted conclusions and future works are exposed in Section 5.

## 2. SEVUE Methodology

A set of 138 images of tomato plants were captured by a Kodak EasyShare *C*913 camera with features corresponding to automatic mode. The image resolution of 320 × 240 pixels was selected to minimize image processing efforts. 30 out of 138 captured images were selected by experts who considered only those captured images of plants showing leaves with early visual symptoms of powdery mildew. Some considerations were made during the image capture sessions, such as the sessions were regularly carried out around midday without considering the climatic conditions (sunny, partly, cloudy, and overcast); the images were captured manually by a person standing in front of and pointing the camera at the vegetation, at a height of approximately 110 cm from the ground and holding the camera at an angle of approximately 45°; no polarizing filter was attached to the camera.

**Figure 1:**
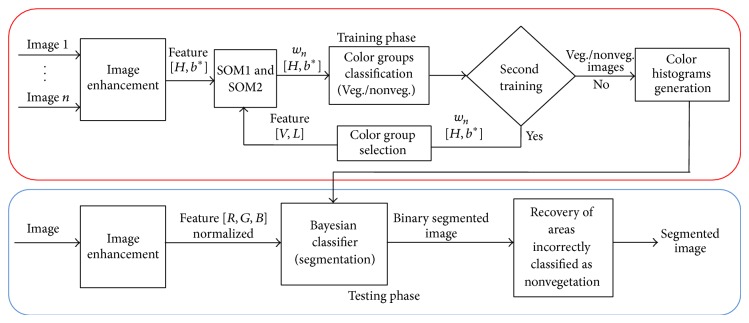
Blocks diagram of the SEVUE methodology. Two phases are performed: training and testing phases. The main processes of SEVUE are the following: an image enhancement to adjust the color and brightness levels of the input images; a color clustering process using SOM1 and SOM2 (SOM2 refines the results of SOM1); a classification process by a Bayesian classifier which segments vegetation from input images; and finally a process for recovering areas incorrectly classified as nonvegetation, which at the output will yield the segmented image.

The SEVUE methodology was tested over the set of 30 images. Due to small statistics of individuals, we applied the *k*-fold cross-validation test aiming at gaining a reasonable estimation of SEVUE methodology accuracy.  Figure 1 illustrates a block diagram that shows the main processes of SEVUE methodology. The next sections will describe in detail the processes of SEVUE by following Figure 1.

### 2.1. Image Enhancement

As a result of the different illumination conditions, the characteristics of light reflected by the objects in the images brought about problems related to different colors and brightness levels. To cope with such problems, it was necessary to adjust the levels of these parameters to homogenize the input images [26], thus achieving a better segmentation of vegetation. For this purpose, the actions described below were applied for each input image.

(1)
A reference image was chosen showing less visual effects due to variable illumination, such as color inconstancy, highlights, shadows, and uneven lighting. The reference image was selected through a visual inspection.
(2)
The reference image and the input image were converted from RGB to YIQ color model.
(3)
The color and brightness of the input image were adjusted using the equations described as follows:
  (1) $$\begin{array}{c} \mu_{\text{im}}=\frac{1}{m\times n}\overset{m\times n}{\underset{n=1}{\sum }}I\left(n\right),\\ \mu_{\text{new}}=\mu_{\text{tar}}-\mu_{\text{im}},\\ I_{\text{new}}=I\left(n\right)+\mu_{\text{new}}, \end{array}$$
 where *I*(*n*) is the intensity value for each channel *Y*, *I*, *Q* from the input image; *μ*_im_ is the mean value for each channel from the input image; *μ*_tar_ is the mean value for each channel *Y*, *I*, *Q* from the reference image with a matrix of size *m* × *n*; *I*_new_ is the new image with an adjustment for both the color and brightness to each channel *Y*, *I*, *Q*.
(4)
The *I*_new_ image is reconverted from YIQ to RGB color model.

**Figure 2:**
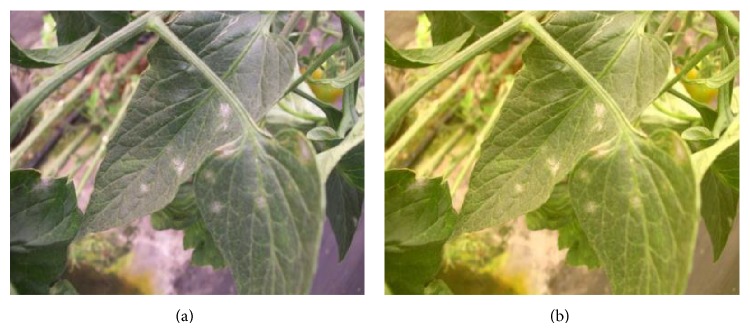
Image enhancement. (a) Original image and (b) image with color and brightness correction.

The resulting image after the enhancement process is an RGB image (see Figure 2). RGB color model is not a good choice for color image processing because it is highly correlated [27], for this reason in the training phase, a color transformation from the RGB color model into HSV and CIE *L* ^*^ *a* ^*^ *b* ^*^ color models are applied to the enhanced image. HSV and CIE *L* ^*^ *a* ^*^ *b* ^*^ are nonlinear color models and are less sensitive to variations of illumination intensity [4]; consequently a more detachable feature space is produced.

### 2.2. SOM Training

A color clustering process is carried out with the set of training images by applying two SOMs. SOM is regarded as a specific region-based image segmentation technique by [28]. SOM combined with *K*-means has achieved better segmentation results in natural color image segmentation compared to JSEG, which is one of the most used techniques for segmentation based on regions of images taken under natural (uncontrolled) conditions [29]. The SOM training generates a map with output neurons, where each neuron represents a color group. The SOM training algorithm applied in SEVUE methodology was proposed by Wu et al. [30], wherein schemes of growing, pruning, and merging of neurons are implemented to find an appropriate number of output neurons automatically. Some considerations were made during the SOM training.

(i) The first SOM considered as input values (features) to the map the *H* and *b* ^*^ components from HSV and CIE *L* ^*^ *a* ^*^ *b* ^*^ color model for each pixel of the image. The components *H* and *b* ^*^ were chosen because they can distinguish between vegetation and background color as is demonstrated in [26].
(ii) The SOM training algorithm is initialized with a number of neurons *N* = 2, which is in accordance with two clusters. The weight vectors for each neuron are initialized with the two first input vectors (*w*_*i*_ = *x*_*i*_, *i* = {1,2}).
(iii) The training algorithm considered a maximum number of neurons to be created, which attempts to represent the color groups on the image effectively. A trial-and-error process was made and best results were observed when the maximum number was defined with a value of 9.
(iv) The winner competition neuron was found applying an heuristic, which is described as follows:
  (2) $$\begin{array}{c} c=\left\{\begin{array}{cc} \text{NULL} & \text{if}\;\;\left(\underset{i}{\text{argmin}}\;v_{i}>\theta \right)\\ \underset{i}{\text{argmin}\;}v_{i} & \text{otherwise} \end{array}\right.\\ v_{i}=D\left(x-w_{i}\right)\;\forall_{i}\in \left\{1,\ldots ,m\right\}, \end{array}$$
 where *v*_*i*_ is the output value of the *i*th neuron with a weight vector *w*_*i*_; *θ* is a threshold with a value of 10 chosen by a trial-and-error process; *x* is an input vector; *D* is the similarity measure between the input vector and the weight vector; in this work the Euclidean distance was considered as measurement. A new neuron is created when *c* = NULL (growing scheme). The input vector *x* is assigned as the weight vector *w* of the new neuron.
(v) The thresholds to decide when to create (growing scheme), eliminate (pruning scheme), and merge neurons were determined by a trial-and-error process. Best results were obtained with a value of 10 for growing and 5 for merging. The elimination of neurons, pruning scheme, was done by deleting that neurons rarely winner (low density) with a frequency less than a predefined percentage, which is decremented in one for each SOM iteration. The initial percentage was defined with a value of 15%.

**Figure 3:**
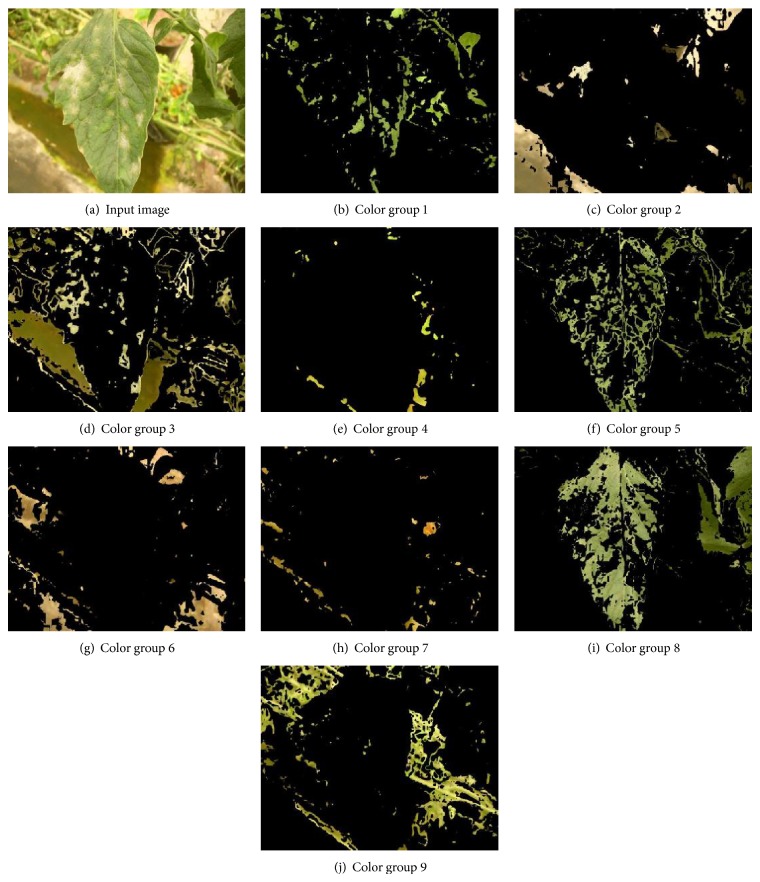
Images generated for the different color groups from an input image. The presented color groups correspond to 2nd iteration of cross-validation.

After the training of the SOM, the set of training images were classified into the 9 color groups (see Figure 3) by assigning each pixel of the images to the color group that corresponds with the minimum Euclidean distance. This process is a presegmentation of the training images based on regions because it consists in grouping pixels of similar intensity levels. This process creates 9 new images for each training image. As observed in Figure 3, the images created for the color groups with a high value in its *H* color component (see Table 1) grouped the green color (vegetation) and those with low values grouped the background color (nonvegetation). The labeling process as vegetation or nonvegetation of the set of new images generated by each color group was possible by using *K*-means. *K*-means clustered into two clusters the color groups by applying the Euclidean square distance as measurement. Then, these clusters were labeled as vegetation or nonvegetation in accordance with their value in the *H* color component by applying a predefined rule (3) as follows:

(3) $$\begin{array}{c} \text{Rule}\;\text{1:}\left\{\begin{array}{cc} \text{if}\;H==\max & \text{then}\;\;C_{i}\left(H,b^{*}\right)=\text{vegetation}\\ \text{else} & C_{i}\left(H,b^{*}\right)=\text{Nonvegetation,} \end{array}\right. \end{array}$$

where *C*_*i*_(*H*, *b* ^*^) is the cluster *i* and *i* = {1,2} (the two result clusters by *K*-means); Max is the maximum value of the *H* channel for the two clusters. Table 1 shows the results for the 10 iterations (*k*) of cross-validation where it is observed that mean values for the vegetation cluster (*V*) correspond to *V*_mean_[57,181] for [*H*, *b* ^*^], respectively.

After classification, it was observed that some color groups labeled as vegetation incorrectly classified background color within its group, or conversely, in groups labeled as nonvegetation some disease or vegetation areas were incorrectly classified. Generally, these classifications errors are presented because of noise information with similar tonalities. For example, in Figure 3 the image corresponding to the color group 3 grouped regions of moldy ground due to similarity with green vegetation. To address these misclassifications, we train a second SOM with the images generated for that color group with the objective of separating those areas and integrating them into their corresponding color group, vegetation or nonvegetation. Through visual inspections to the set of images, it was observed that the color groups with misclassifications presented values [*H*, *b* ^*^] lower than the mean value for vegetation *V*_mean_[57,181]. Considering those observations, it was possible to implement a procedure to select automatically the color group of nonvegetation or vegetation to be trained again.

(1)
Search in the vegetation color groups *w*_*ik*_[*H*, *b* ^*^] < *V*_mean_[57,181], where *i* is the color group, *i* = {1,…, 9}, and *k* is the fold in the cross-validation *k* = 1,…, 10.
(2)
If some color group is selected, then
(2.1)
the color group with the maximum Euclidean distance to *V*_mean_ is chosen to be trained,
(2.2)
search in the rest of vegetation color groups *w*_*ik*_[*H*, *b* ^*^] where *H* < 57 and relabel them as nonvegetation.
(3)
else
(3.1)
search in the nonvegetation color groups *w*_*ik*_[*H*, *b* ^*^] < *V*_mean_[57,181]
(3.2)
if some color group is selected, then
(3.2.1)
the color groups with the minimum Euclidean distance to *V*_mean_ is chosen to be trained.

**Figure 4:**
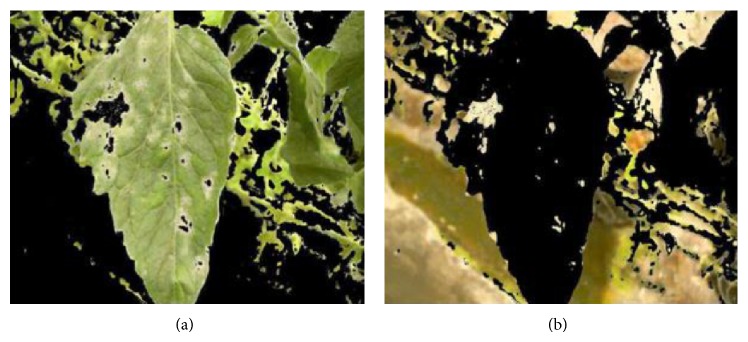
The resultant images by joining the different color groups. (a) The resultant image for the color groups labeled as vegetation and (b) the resultant image for the color groups labeled as nonvegetation.

This procedure is repeated for each one of the 10-fold. The color groups selected by the procedure are highlighted in Table 1. For example, for *k* = 2 the color group *w*_3_ was selected to be trained again and the color group *w*_4_ was relabeled as nonvegetation. All images generated for the selected color group assume the role of input to the second SOM training. The training considerations for the second map are the same as presented at the beginning of this section, except that, in this case, we considered the *V* channel from the HSV color model and *L* from the CIE *L* ^*^ *a* ^*^ *b* ^*^ color model as the input values to the map because they showed better discrimination between vegetation and background with similar tonalities to vegetation (moldy ground). The *V* and *L* color components were chosen based on the observations of the resulting images due to the fact that it was not possible to quantify the segmentation performance between color components because the manual segmentation of the background was not considered. As final step of the color clustering process, all the color groups images labeled as vegetation are got together to create a new set of images (see Figure 4(a)). The same process is applied to the color group images labeled as nonvegetation (see Figure 4(b)). Therefore the new set of images consists of 30 vegetation images and 30 nonvegetation images which will be used as knowledge by the supervised classifier (Bayesian classifier) for vegetation segmentation purposes. The next section describes details about the Bayesian classifier.

### 2.3. Image Segmentation by the Bayesian Classifier

The vegetation segmentation process is carried out by a Bayesian classifier, performing a pixel level classification of the input images for labeling each pixel as vegetation or nonvegetation. Bayesian reasoning is based on the assumption that optimal decisions can be made by relating probability distributions with observed data. When these probabilities are not known a priori they are often estimated by a training process, where examples are incorporated into an algorithm and a supervisor determines the different classes. The set of vegetation and background images created by the SOM clustering process are considered as examples for training the classifier; in our case the classes were determined automatically by the SEVUE methodology. The conditional probabilities are estimated by creating two color histogram models (vegetation, nonvegetation) from the examples images in the training process. The histogram represents the relative frequency of each combination (*rgb*) in the image. For classification purposes, the histogram counts are converted into discrete probability distributions as follows:

(4) $$\begin{array}{c} P\left(rgb\right)=\frac{c\left(r,g,b\right)}{T_{c}}, \end{array}$$

where *c*(*r*, *g*, *b*) represents the count in the histogram bin associated with the (*r*, *g*, *b*) color combination and *T*_*c*_ is the total count obtained by summing the counts in all of the bins. We formed the histograms with a bins number of 64 × 64 × 64. Given vegetation and nonvegetation histograms, we can compute the probability that a given color value (a (*r*, *g*, *b*) combination) belongs to the vegetation (*v*) and nonvegetation (~*v*) classes using the Bayes theorem, described as

(5) $$\begin{array}{c} P\left(v\;|\;rgb\right)=\frac{P\left(rgb\;|\;v\right)P\left(v\right)}{P\left(rgb\;|\;v\right)P\left(v\right)+P\left(rgb\;|\;\sim v\right)P\left(\sim v\right)}, \end{array}$$

where the conditional probabilities *P*(*rgb*∣*v*) and *P*(*rgb*∣ ~ *v*) and a priori probabilities *P*(*v*) and *P*(~*v*) are directly computed from the vegetation and nonvegetation histograms, respectively,

(6) $$\begin{array}{c} P\left(rgb\;|\;v\right)=\frac{v\left(r,g,b\right)}{T_{v}},\\ P\left(rgb\;|\;\sim v\right)=\frac{nv\left(r,g,b\right)}{T_{nv}}, \end{array}$$

where *v*(*r*, *g*, *b*) is the pixel count contained in bin (*r*, *g*, *b*) of the vegetation histogram, *nv*(*r*, *g*, *b*) is the equivalent count from the nonvegetation histogram, and *T*_*v*_ and *T*_*n*_ *v* are the total counts contained in the vegetation and nonvegetation histograms, respectively.

A pixel is classified as vegetation if

(7) $$\begin{array}{c} P\left(v\;|\;rgb\right)\geq \theta , \end{array}$$

where 0 ≤ *θ* ≤ 1 is a threshold. Alternatively, a faster way is to apply the following rule

(8) $$\begin{array}{c} P\left(rgb\;|\;v\right)\geq P\left(rg\;|\;\sim v\right). \end{array}$$

### 2.4. Recovering Areas Incorrectly Classified as Nonvegetation

**Figure 5:**
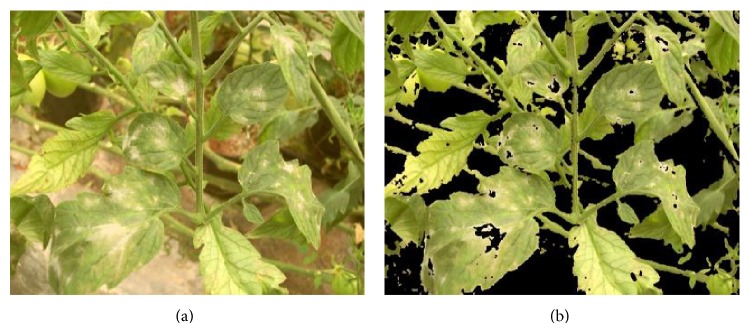
(a) Original image and (b) same image after applying Bayesian classifier.

In applications of vegetation segmentation in natural and complex environments, the color has yielded efficient results as shown by methods such as CIVE [21] and excess-green [22], among others. However, some ambiguities related to color pixels may arise, due to similarities between the disease color and the background color [4], when the segmentation of diseased vegetation is being carried out. In this work, a color ambiguity problem appears in the images because of color similarities between the background and the visual symptoms of powdery mildew disease observed in the leaves. After applying the Bayesian classification, it was observed that some lesion areas of the images were labeled as nonvegetation (see Figure 5), which is due to the fact that the similarity of color tones causes the SOM to integrate both the background and the disease areas into the same color group.

This work proposes the extraction and analysis of contours from the binary image generated after the Bayesian classification for recovering areas incorrectly classified as nonvegetation. The algorithm proposed in [31] was used for the contour extraction. The basic concept of this algorithm consists of tracking the edges by considering a topological analysis. Therefore, the relationship between inner and outer edges is extracted.

**Figure 6:**
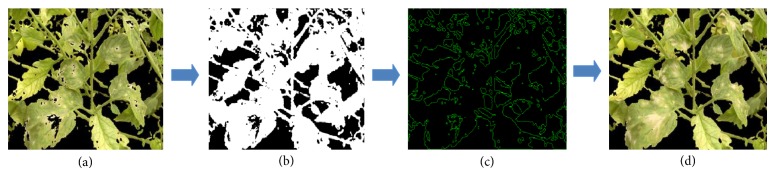
A process that extracts and selects contours which encloses disease areas. (a) Image segmented with the Bayesian classifier, (b) binary image, (c) contour extraction, and (d) recovering of contours corresponding to vegetation/disease areas and noise elimination.

The most important contours for the purpose of this work were those that represent the inner edges. Once contours were extracted, the next step is to select and analyze the contours whose measurement in pixels is less than a predefined threshold. The process is described in Figure 6.

Powdery mildew is manifested in its initial stage with small whitish spots on leaves. For this reason, it was considered that small contours that correspond to areas surrounded by pixels labeled as vegetation probably are contours of disease areas. After testing multiple values of thresholds, it was considered that contours with a pixel count less than 300 pixels and larger than 20 pixels represent diseased areas. It was inferred that contours larger than 300 pixels correspond to vegetation regions or background; therefore they are not analyzed.

A contour with a pixel count less than 20 pixels is considered as noise. The area that these small contours enclose is labeled as vegetation or nonvegetation by analyzing its neighboring pixels from the binary image, counting those pixels labeled with 1 (vegetation neighbor pixel) and with 0 (nonvegetation neighbor pixel). If the count for neighbor pixels labeled as vegetation is bigger than the count for nonvegetation pixels, then the contour area is labeled as vegetation, otherwise as nonvegetation.

In the case of contours larger than 20 pixels, the analysis of the area that encloses the contour was made by considering its standard deviation (*S*) for both *H* and *b* ^*^ channels, which is obtained by

(9) $$\begin{array}{c} S={\left(\frac{1}{n-1}\overset{n}{\underset{i=1}{\sum }}{\left(g_{i}-\bar{g}\right)}^{2}\right)}^{1/2}, \end{array}$$

where *n* is the area (number of pixels) that encloses the contour, *g*_*i*_ represents the *H* or *b* ^*^ value of the pixel *i*, and $\bar{g}$ is the mean value of *H* or *b* ^*^ in the area enclosed by the contour. The rule for labeling the contour area as vegetation or nonvegetation is described as follows:

(10) $$\begin{array}{c} \text{Rule}\;\text{2:}\left\{\begin{array}{cc} \text{if }\left(S_{H}>\theta ,S_{b^{*}}>\theta \right) & \text{then }C_{z}=\text{vegetation}\\ \text{else} & C_{z}=\text{Nonvegetation,} \end{array}\right. \end{array}$$

where *C*_*z*_ is the analyzed contour and *θ* is a threshold with a value of 2.4. The value was chosen after carrying out experiments with multiple values and the best results were observed with this threshold.

## 3. Assessing the Performance of SEVUE Methodology

The performance assessment of the vegetation segmentation methodology proposed in this work is quite difficult because the uncontrolled illumination and background conditions bring about highly complex images to be analyzed. Some techniques that have been used for this purpose consist of comparing an image that has been segmented manually with the same image but segmented using a proposed methodology [6, 22, 25]. The procedure used in this work to assess the methodology performance is based on the approach mentioned before which was proposed by Camargo and Smith [6]. They compared the two images from two perspectives, the first one by analyzing all the pixels from the image *z*  (11) and the second one by analyzing the pixels that are considered as disease *d*  (12). Consider the following:

(11) $$\begin{array}{c} z=\overset{m}{\underset{i=1}{\sum }}\overset{n}{\underset{j=1}{\sum }}I\left(i,j\right), \end{array}$$

(12) $$\begin{array}{c} d=\overset{m}{\underset{i=1}{\sum }}\overset{n}{\underset{j=1}{\sum }}I\left(i,j\right)==1. \end{array}$$

The manual segmentation was done by overlapping a grid on the image; then each box was evaluated and labeled according to a color schema; white(1) was used to represent a disease region and black(0) was used to represent a nondiseased region. The same process was done for labeling vegetation regions.

The images used by Camargo and Smith [6] correspond to a single leaf. They focused on the disease areas without considering complex background. In our work we did not analyze all the pixels of the image, but only those areas of the image that correspond to leaves with lesion regions which are localized in the foreground. Therefore, *z*  (13) was modified to analyze only the pixels labeled manually as vegetation:

(13) $$\begin{array}{c} z=\overset{m}{\underset{i=1}{\sum }}\overset{n}{\underset{j=1}{\sum }}I\left(i,j\right)==1. \end{array}$$

The manual segmentation carried out in this work consisted of marking separate contours on the image in areas considered either as vegetation or as diseased; the pixels into the area enclosed by the contour were labeled with the value of 1. The set of pixels manually labeled either as vegetation or as disease are represented by (*p*_*v*_) or (*p*_*d*_), respectively, and those automatically labeled using the SEVUE methodology are represented by (*q*_*v*_) or (*q*_*d*_). Then the comparison was made according to the following:

(14) $$\begin{array}{c} z=\overset{m}{\underset{i=1}{\sum }}\overset{n}{\underset{j=1}{\sum }}\left(\left(p_{v}\left(i,j\right),q_{v}\left(i,j\right)\right)==1\right),\\ d=\overset{m}{\underset{i=1}{\sum }}\overset{n}{\underset{j=1}{\sum }}\left(\left(p_{d}\left(i,j\right),q_{d}\left(i,j\right)\right)==1\right). \end{array}$$

The percentage of missegmentation of disease (*d*_*e*_) and vegetation (*z*_*e*_) areas is represented by the following equation:

(15) $$\begin{array}{c} z_{e}=\frac{\left(\sum_{i=1}^{m}\sum_{j=1}^{n}\left(\left(p_{v}\left(i,j\right)!=q_{v}\left(i,j\right)\right)\right)\times \left(m\times n\right)\right)}{100},\\ d_{e}=\frac{\left(\sum_{i=1}^{m}\sum_{j=1}^{n}\left(\left(p_{d}\left(i,j\right)!=q_{d}\left(i,j\right)\right)\right)\times \left(m\times n\right)\right)}{100}. \end{array}$$

## 4. Results and Discussion

The *K*-fold cross-validation technique was applied in this work with the goal of quantitatively assessing the performance of the proposed methodology (SEVUE). The value of *k* was considered in 10-fold that correspond to 10 iterations. A set of 30 images was used. Most of the images presented leaves with areas that corresponded to a visual symptom of disease. The images were manually segmented by three users, who separately marked on the images the regions that they considered as vegetation or as disease. The users segmented only those regions in foreground due to the fact that these provide relevant information from a practical point of view, as opposed to regions at the rear of the scene because these were blurred.

The most relevant results to be assessed are related to aspects such as the segmentation rate of diseased and healthy vegetation in tomato images and the recuperation of these disease or vegetation areas incorrectly classified as nonvegetation after the application of the Bayesian classification.

### 4.1. Segmentation of Healthy and Diseased Vegetation

An initial assessment was made to determine the accuracy of the SEVUE methodology for segmenting greenness from color images. The methodology results were assessed up to the Bayesian classification process shown in Figure 1, without considering the process of extraction and analysis of contours for the recuperation of incorrectly classified areas.

The vegetation segmentation results obtained by the SEVUE methodology were assessed by comparing them with the manual segmentation (as described in Section 3) done by each user. Additionally, two methods were implemented and assessed for vegetation segmentation: the two most used color index methods, CIVE and ExG-ExR. The results of the mentioned methods were compared with those yielded by the SEVUE methodology.

The ExG-ExR is calculated using (16), from [22], which are described as

(16) $$\begin{array}{c} \text{ExG}=2g-r-b\\ \text{ExR}=1.4r-g, \end{array}$$

where *r*, *g*, and *b* are the normalized components and they are determined as follows:

(17) $$\begin{array}{c} r=\frac{R^{*}}{R^{*}+G^{*}+B^{*}},\;\;g=\frac{G^{*}}{R^{*}+G^{*}+B^{*}},\\ b=\frac{R^{*}}{R^{*}+G^{*}+B^{*}}. \end{array}$$

*R* ^*^, *G* ^*^ and *B* ^*^ are the values of the color channels of RGB image, which have also been previously normalized as a range from 0 to 1; they are defined as follows:

(18) $$\begin{array}{c} R^{*}=\frac{R}{R_{\max}},\;\;G^{*}=\frac{G}{G_{\max}},\;\;B^{*}=\frac{B}{B_{\max}}, \end{array}$$

and *R*_max⁡_, *G*_max⁡_, *B*_max⁡_ = 255 are the maximum total value for each primary color. The color index CIVE is calculated using the following equation from [21]:

(19) $$\begin{array}{c} \text{CIVE}=0.441R-0.811G+0.385B+18.787. \end{array}$$

After applying the color indices, the output images are gray level images. An Otsu threshold method was adopted to convert each CIVE image to a binary image and a zero threshold in the case of result image from ExG-ExR.

**Figure 7:**
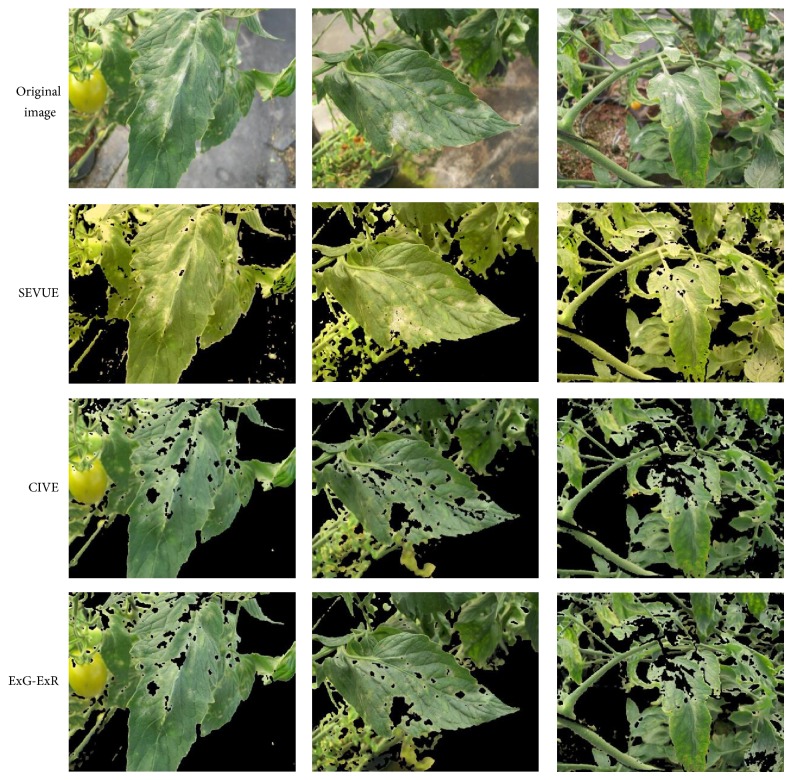
Example results of segmented images by the three methods being compared.

Images segmented by the three methods are shown in Figure 7. Compared with CIVE and ExG-ExR, the SEVUE methodology results showed that segmentation rate is superior by incorporating some disease areas that are not segmented by these methods. Also ripe fruits are removed and less background information is included.

Table 2 shows the average for matching and missegmentation between the manual segmentation performed by each one of the three users and the automatic segmentation performed by the three methods. The column Match (matching) of Table 2 shows the averages of the matching results between the vegetation areas (including disease areas) manually segmented by each user (*S*1, *S*2, *S*3) with the vegetation areas automatically segmented by SEVUE (the proposed methodology), CIVE, and ExG-ExR; meanwhile, the column Miss. (missegmentation) shows the missegmentation results between the disease areas manually segmented and those automatically segmented for each method (only areas of disease). The percentages shown in the table are the average results of the cross-validation after 10 iterations for each user and for each method.

It is observed from Table 2 that the average difference for matching between the manual and automatic segmentation was 5.2% for SEVUE, 24.2% for CIVE, and 11.6% for ExG-ExR. The smallest average difference for matching was obtained by SEVUE and also the smallest average missegmentation with 28.69%, considerably better than the other methods.

### 4.2. Recovery of Areas Incorrectly Classified as Nonvegetation

**Figure 8:**
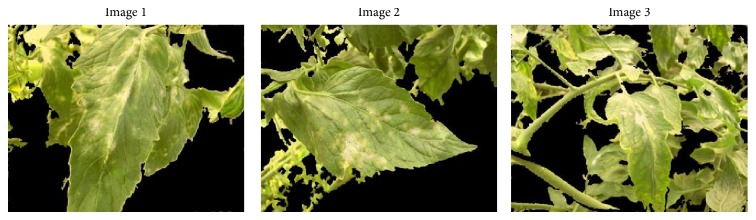
Example of resulting images after applying the process for recovering vegetation and disease areas incorrectly classified as nonvegetation.

The second aspect to be assessed is related to the obtained results after executing the process aimed at recovering vegetation and disease areas that were not properly segmented. This process consists in the extraction and analysis of contours, as described in Section 2.4.  Figure 8 shows some examples of resulting images after applying the process for recovering disease and vegetation areas incorrectly classified as nonvegetation to the same images presented in the previous section (Figure 7). Derived from a visual inspection of the image results some conclusions were extracted, which are described below.

- The SEVUE methodology recovers a larger area of diseased regions.
- Small isolated areas classified as vegetation, and considered as noise, were accurately removed by SEVUE.
- We have to highlight that in spite of the fact that the changing illumination conditions and the background areas with similar color as vegetation could have affected the vegetation segmentation results in the image, these results were considerably robust as shown before.
- A limitation of the methodology is related to the nonelimination of green fruits from the images and also to small areas belonging to background labeled by the algorithm as vegetation.

The results presented in Table 3, which have been obtained after the execution of all of the process of the SEVUE methodology, show the matching and missegmentation percentage between the manual and automatic segmentation. These results correspond to each set of images that were segmented by the three users (*S*1, *S*2, *S*3).

The percentages are represented by the average results of each set of images belonging to the iterations of the cross-validation. It was observed that the SEVUE methodology obtained better results, with a missegmentation average of 8.62% (from Table 3), compared to CIVE with an average of 61.9% (from Table 2) and ExG-ExR with 52.7% (from Table 2). It is observed, from Table 3, that the average difference for missegmentation, between the manual and automatic segmentation, was improved by SEVUE with an average of 20% after applying the process for recovering areas incorrectly classified as nonvegetation.

The Wilcoxon signed rank test assessed the reliability of the SEVUE results taking into consideration the percentages of matching and missegmentation for the whole set of training images (30 images). A statistical significance level of 5% was considered. The analysis shows that the *P* values for the missegmentation of disease areas are less than 0.05 and hence, for all cases, null hypothesis is rejected and alternative hypothesis is accepted to confirm that there is a significant difference between the proposed methodology SEVUE and the methods proposed by CIVE and ExG-ExR.

## 5. Conclusions

This work dealt with a methodology for segmenting healthy and diseased vegetation from images of tomato plants under uncontrolled outdoor illumination conditions. These conditions make the problem a complex task to be confronted. However, in spite of using vision equipment with technical limitations, we proposed in this work a methodology that copes with this problem yielding better results compared with other previously reported methods. The methodology includes an enhancement process of the captured image quality, coping in this way with the unpredictable effects of illumination. In addition, an unsupervised learning method (SOM) whose function consisted in separating the color images previously enhanced in vegetation and nonvegetation color groups. The methodology also includes a supervised method (Bayesian classifier) whose a priori knowledge was the vegetation and nonvegetation colors extracted from images by the SOM, and its function consisted in the vegetation segmentation from images. The proposed procedure for recovering areas incorrectly classified as nonvegetation, after the application of the Bayesian classification and the inclusion of a second SOM training into the methodology, demonstrated its capacity to achieve a better segmentation rate than the performance yielded by the color index methods CIVE and ExG-ExR. A comparison process that confronted the performance of the proposed methodology with images manually segmented by users yielded good results. The average difference of the matching between the manual and automatic vegetation segmentation (including disease areas) was 2.2% for SEVUE, 11.6% for ExG-ExR, and 24.2% for CIVE. The average of missegmentation of disease areas was 8.62% for SEVUE, 52.7% for ExG-ExR, and 61.9% for CIVE. A nonparametric statistical hypothesis test using the Wilcoxon signed rank sum test demonstrated that missegmentation results of the proposed methodology SEVUE were significantly better compared with CIVE and ExG-ExR. Summarizing, it is concluded that the developed methodology is relatively robust vis-à-vis the outdoor illuminations and stable for applications under uncontrolled environments. We aimed at presenting the methodology SEVUE in practical terms. Therefore, we consider that this methodology can be replicated by the interested users. Some important future works to be performed are (1) to use the segmented images in applications of disease recognition; (2) to assess the performance of the proposed methodology in the segmentation of diseases areas associated with other vegetables.

## Figures and Tables

**Table 1:** Weights of the color groups.

*K*	Label	Clusters *C*_*ik*_(*H*, *b* ^*^)	Neurons								
			*w*_1*k*_(*H*, *b* ^*^)	*w*_2*k*_(*H*, *b* ^*^)	*w*_3*k*_(*H*, *b* ^*^)	*w*_4*k*_(*H*, *b* ^*^)	*w*_5*k*_(*H*, *b* ^*^)	*w*_6*k*_(*H*, *b* ^*^)	*w*_7*k*_(*H*, *b* ^*^)	*w*_8*k*_(*H*, *b* ^*^)	*w*_9*k*_(*H*, *b* ^*^)
1	NV	**(41.26, 167.16)**	(39.14, 159.57)	—	—	—	(35.43, 171.94)	(49.21, 169.97)	—	—	—
	V	**(59.16, 178.98)**	—	(52.24, 198.69)	(51.38, 181.41)	**(56.37, 169.22)**	—	—	(64.14, 182.73)	(67.90, 175.4)	(62.93, 166.40)

2	NV	**(38.17, 163.41)**	—	(39.71, 155.54)	—	—	—	(36.63, 171.28)	(41.28, 187.83)	—	—
	V	**(58.06, 179.94)**	(69.20, 177.63)	—	**(54.20, 169.54)**	(52.32, 198.74)	(66.75, 174.24)	—	—	(63.90, 167.44)	(58.74, 184.15)

3	NV	**(45.41, 164.34)**	—	—	—	(33.76, 191.09)	(35.58, 167.74)	—	(42.55, 156.83)	—	—
	V	**(52.73, 187.60)**	(58.09, 168.46)	(53.88, 192.54)	(60.34, 183.13)	—	—	**(49.08, 180.95)**	—	(68.03, 175.43)	(51.28, 202.48)

4	NV	**(47.18, 193.35)**	—	(35.75, 169.29)	—	(42.50, 156.78)	—	—	(36.97, 197.03)	—	—
	V	**(53.84, 170.14)**	(63.75, 168.50)	—	(59.93, 181.35)	—	(52.29, 184.13)	(52.27, 198.88)	—	(68.15, 175.92)	**(52.96, 168.98)**

5	NV	**(44.73, 167.02)**	—	—	—	—	(45.84, 159.30)	(46.51, 173.27)	(34.92, 171.31)	(36.41, 162.74)	—
	V	**(55.69, 188.93)**	(64.49, 180.24)	(53.44, 191.91)	**(53.56, 181.08)**	(59.96, 168.45)	—	—	—	—	(51.29, 202.50)

6	NV	**(37.43, 172.39)**	(33.93, 191.49)	—	(42.66, 156.74)	—	—	(35.71, 168.93)	—	—	—
	V	**(58.74, 180.29)**	—	(62.55, 184.94)	—	**(55.72, 172.1)**	(51.12, 182.61)	—	(52.23, 198.66)	(62.76, 166.7)	(68.04, 176.7)

7	NV	**(49.33, 167.52)**	(37.29, 171.13)	(52.38, 191.73)	—	—	—	—	(40.96, 155.81)	(48.47, 175.31)	(51.29, 202.50)
	V	**(58.32, 187.88)**	—	—	**(56.08, 169.29)**	(60.81, 181.56)	(68.82, 175.71)	(63.82, 166.05)	—	—	—

8	NV	**(44.62, 197.96)**	—	—	—	(35.75, 169.29)	(42.5, 156.78)	(36.97, 197.03)	—	—	—
	V	**(53.33, 172.42)**	(63.75, 168.5)	(52.29, 184.13)	(52.27, 198.88)	—	—	—	(59.93, 181.35)	(67.57, 177.05)	**(51.53, 169.82)**

9	NV	**(47.08, 169.14)**	—	(47.16, 178.72)	(39.06, 159.53)	(35.41, 171.93)	—	**(52.34, 168.30)**	—	—	(51.30, 202.50)
	V	**(58.40, 187.33)**	(53.35, 190.97)	—	—	—	(59.68, 180.49)	—	(69.28, 175.36)	(61.41, 167.20)	—

9	NV	**(44.30, 167.84)**	—	(43.41, 157.05)	(52.22, 156.84)	**(53.72, 168.62)**	—	(41.79, 165.66)	(30.38, 191.05)	—	—
	V	**(66.63, 182.29)**	(66.04, 198.96)	—	—	—	(64.26, 175.46)	—	—	(66.78, 186.03)	(69.44, 168.72)

**Table 2:** Matching and missegmentation results.

User	SEVUE		CIVE		ExG-ExR	
	Match.	Miss.	Match.	Miss.	Match.	Miss.
S1	95.181	27.003	75.906	58.391	88.796	50.284
S2	94.821	30.920	76.267	66.213	88.539	54.895
S3	94.287	28.155	75.149	61.282	87.726	53.171
Avg.	**94.76**	**28.69**	**75.77**	**61.96**	**88.35**	**52.78**

**Table 3:** Matching and missegmentation results.

Iter.	S1		S2		S3		Avg.	
	Match.	Miss.	Match.	Miss.	Match.	Miss.	Match.	Miss.
1	90	52.5	90.22	37.54	90.82	36.42	90.35	42.15
2	98.33	8.43	98.02	6.38	97.46	5.6	97.93	6.8
3	99.84	0.15	99.38	0	98.61	0.15	99.28	0.1
4	99.07	0.21	98.85	0.03	98.78	1.09	98.9	0.44
5	98.74	6.5	98.22	15.26	97.64	7.41	98.2	9.72
6	94.77	10.44	95.4	11.11	93.42	10.38	94.53	10.64
7	97.69	9.96	97.6	15.96	96.68	17.45	97.33	14.46
8	99.66	0.03	99.27	0.06	99.42	0.07	99.45	0.05
9	98.67	0	98.78	0	98.41	3.24	98.62	1.08
10	99.44	0.04	97.97	0	98.75	2.3	98.72	0.78

Total	97.62	8.83	97.37	8.63	97	8.41	**97.33**	**8.62**
